# Tuberculosis Topics

**Published:** 1920-11-06

**Authors:** 


### Tuberculosis Topics.

IN his annual report for the year ended March 31, 1920, Dr. Trimble, the chief tuberculosis officer of Belfast, gives some interesting statistics. The deaths from tuberculosis were 1,122, and of these 853 died of the pulmonary form. This was a reduction on last year by 198, the calculated ratio being 2.1 per thousand living in 1919, as against 2.7 for 1918. The number of new patients in the year was about 2,520, only 96 being bone and joint cases. Females still were in excess of male patients, this being probably a persistence of a war phenomenon, although the difference was not so great as in former years, owing to the fact that many discharged soldiers were under treatment for tuberculosis due to war service. Pulmonary tuberculosis had increased from 77 per cent. in 1918 to 82 per cent. in 1919, whilst non-pulmonary forms had diminished. As for increase in accommodation, the purchase by the Corporation of the Graymount estate for conversion into a hospital for osseous and non-pulmonary forms of the disease, and also the effort to establish an open-air school, these are steps in the right direction. We might comment, however, that few houses will make even temporary hospitals on the Alton lines without great alteration, the difficulty being to get beds out and in quickly. The need for an open-air school is obvious; but, prevention being better than cure, the open-air planning of all schools would be better still.

